# The Leaf Wettability of Various Potato Cultivars

**DOI:** 10.3390/plants9040504

**Published:** 2020-04-14

**Authors:** Ewa Papierowska, Jan Szatyłowicz, Stanisław Samborski, Joanna Szewińska, Elżbieta Różańska

**Affiliations:** 1 Water Centre, Warsaw University of Life Sciences – SGGW, ul. Jana Ciszewskiego 6, 02-766 Warsaw, Poland; 2 Institute of Environmental Engineering, Warsaw University of Life Sciences – SGGW, ul. Nowoursynowska 159, 02-776 Warsaw, Poland; jan_szatylowicz@sggw.pl; 3 Institute of Agriculture, Warsaw University of Life Sciences – SGGW, ul. Nowoursynowska 159, 02-776 Warsaw, Poland; stanislaw_samborski@sggw.pl; 4 Institute of Biology, Warsaw University of Life Sciences – SGGW, ul. Nowoursynowska 159, 02-776 Warsaw, Poland; joanna_szewinska@sggw.pl (J.S.); elzbieta_rozanska@sggw.pl (E.R.)

**Keywords:** potato cultivar, contact angle, sessile drop method, potato leaf wettability, *Phytophthora infestans*

## Abstract

Leaf wettability has an impact on a plant’s ability to retain water on its leaf surface, which in turn has many environmental consequences. In the case of the potato leaf (*Solanum tuberosum* L.), water on the leaf surface may contribute to the development of a fungal disease. If fungal disease is caused, this may reduce the size of potato harvests, which contribute significantly to meeting global food demand. The aim of this study was to assess the leaf wettability of five potato cultivars (i.e., Bryza, Lady Claire, Rudawa, Russet Burbank, Sweet Caroline) in the context of its direct and indirect impact on potato yield. Leaf wettability was assessed on the basis of contact angle measurements using a sessile drop method with an optical goniometer. For Bryza and Rudawa cultivars, which showed, respectively, the highest and the lowest contact angle values, light microscopy as well as scanning electron microscopy analyses were performed. The results of the contact angle measurements and microscopic image analyses of the potato leaf surfaces indicated that the level of wettability was closely related to the type of trichomes on the leaf and their density. Therefore, higher resistance of the Rudawa cultivar to biotic stress conditions could be the result of the presence of two glandular trichome types (VI and VII), which produce and secrete metabolites containing various sticky and/or toxic chemicals that may poison or repel herbivores.

## 1. Introduction

Potato (*Solanum tuberosum* L.), the world’s third most important food crop in terms of human consumption, makes a significant contribution to meeting global food demands and also to trade, with enormous economic benefits. The world’s total potato production is increasing and was estimated at about 368 million tonnes in 2018. In 2005, for the first time, the developing world’s potato production exceeded that of the developed world; currently, about 38% of production takes place in China and India. Poland is ranked eighth in terms of global potato production. However, from 1961 to 2017, the total area used for potato cultivation in Poland decreased by 88%, with a simultaneous increase in yield (in kg ha^−1^) of about 74% [1]. This increase in yield was associated, among other factors, with an increase in the amount of fertilizers and plant protection products used. In 2016, the amount of pesticides used in Poland reached 24,462.5 tonnes, and this value has increased almost 5-fold since 1990 [1]. The increase in yield is also associated with the amount and quality of water used for irrigation, but this depends on irrigation scheduling and the method of application of this water [2].

The potato leaf surface is very sensitive to all types of stress [3]. The plant can produce more wax under water stress, which is usually more hydrophobic than the leaf surface itself [4]. Kim et al. [5] demonstrated a 30% increase in total wax as a response to drought in most sesame genotypes (*Sesamum indicum* L.), and Kosma et al. [6] also showed an increase in wax per unit area from 32% to 80%, mainly due to the increase in wax alkanes for Arabidopsis (*Arabidopsis thaliana*) under stress. It is important that, apart from waxes, leaf hydrophobicity is determined by other factors including the presence of hair, nerves, damage, etc. [7]. Leaf hydrophobicity is most often determined on the basis of contact angle measurement [4,8,9]; indeed, contact angle is often treated as a bioindicator of urban habitat quality [10] and as a bioindicator of environmental pollution. Hydrophobic polycyclic aromatic hydrocarbons can settle on young leaves, resulting in an increase in their hydrophobicity, or can reduce hydrophobicity through long-term leaf exposure to air pollution [11]. 

Potato is very sensitive to water stress [12,13,14], and its cultivars are known to differ response to drought conditions due to their contrasting canopy architecture, stem versus leaf type [15], and specific leaf area [16]. Water stress also reduces the surface area of potato leaves and reduces photosynthesis per unit area [17]. 

Water droplets (rain, fog, etc.) are repelled from hydrophobic leaf surface, and water does not spread on the leaf but rather flows down the surface to feed the root layer [18,19]. The opposite can be said for leaves with wettable surfaces, on which a droplet of water behaves differently, i.e., spreads on the leaf and stays on the surface [20]. The presence of a water droplet on a leaf’s surface can slow down the photosynthesis process, as carbon dioxide diffuses more slowly in water than in air [8]. In addition, the persistence of water on leaf surfaces may contribute to the development of the fungal disease *Phytophthora infestans* (Mont.) de Bary [21,22], which may cause serious yield reduction of potato in unprotected plantations in years with cool and humid environmental conditions. Management of *P. infestans* with chemical fungicides is usually the most successful agricultural practice. However, due to environmental and social reasons, modern approaches require a reduction of fungicide inputs and use of potato cultivars with higher resistance to late blight [23]. Fungicides of a diverse chemical nature are applied to potato leaves, which show high variation in their morphology. This observation has raised questions regarding whether potato leaf structure might impact the effectiveness of fungicide sprays against *P. infestans*. Papierowska et al. [24] showed that leaf wettability and leaf surface structures, in particular the presence of trichomes, also have an impact on the drop splash phenomenon, which could potentially also affect the spread of potato diseases [25].

There are many entries in the literature regarding crop leaf wetting, including crops from the Solanaceae family: tomatoes, peppers, and eggplant [26]; however, in the case of the potato, there is a gap in the research. Therefore, the aim of this study was to assess the wettability of various cultivars of *Solanum tuberosum* L. Moreover, a microscopic analysis of the internal structure of the leaf blades was carried out to explain the possible cultivar differences in leaf wettability and potential resistance to biotic stress conditions.

## 2. Results

### 2.1. Contact Angle Measurements

**Figure 1:**
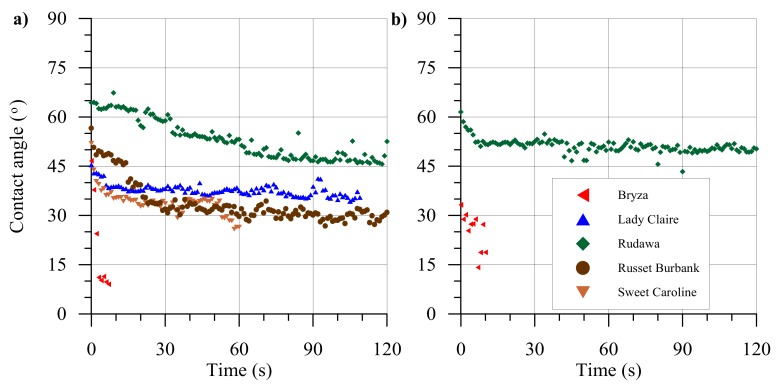
Examples of contact angle measurements for a single distilled water drop on leaves of five potato cultivars over 120 s. Leaves were collected in 2018 (**a**) and in 2019 (**b**).

Contact angles were measured over 120 s for single droplets, the results are presented in Figure 1. For all examined cultivars, a decrease in the contact angle values as a non-linear function of time was observed. The fastest (in less than 10 s) spreading was recorded for a droplet of distilled water placed on the Bryza leaf cultivar; on the Rudawa leaf cultivar, it remained on the surface for a greater amount of time than the 120 s period of measurement, i.e., >120 s.

**Figure 2:**
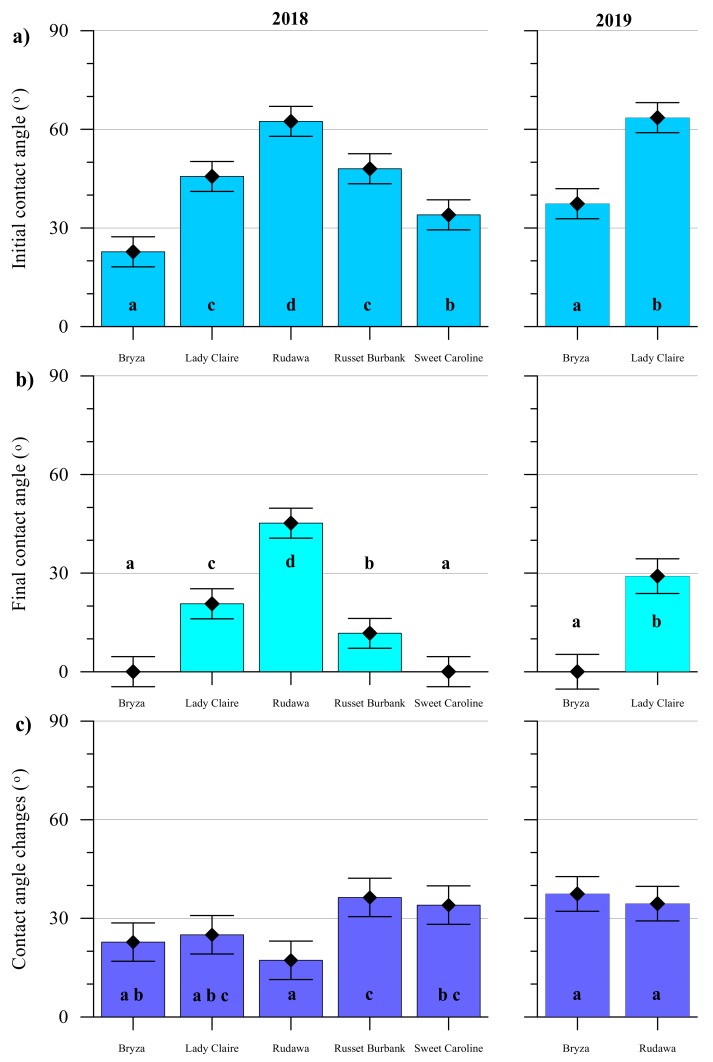
Contact angle measurement results for five potato cultivars in 2018 and two in 2019: (**a**) initial contact angle; (**b**) final contact angle; (**c**) change of contact angle values in time. The same letters denote homogenous groups based on two-factor analysis of variance at a 0.05 probability level.

The measured contact angle values divided into the initial contact angle, the final contact angle, and the change of the angle value over the time of 120 s, as well as the results of the analysis of the variances between cultivars, are presented in Figure 2. In both years, the highest contact angle values were found for the Rudawa cultivar. For this cultivar, both the initial contact angle (i.e., measured after 1 s) and the final contact angle (measured after 120 s) were the highest among all the cultivars. In fact, this cultivar was characterized by the smallest change in the contact angle value. The droplet behaved the most stably during the measurement, i.e., for 2 minutes, compared to the other cultivars. In turn, the highest contact angle change over time was observed for Russet Burbank cultivar. The lowest contact angle values were found for the Bryza cultivar (initial contact angle < 30°). In the case of this cultivar, the droplet spread over the leaf surface quickly within the 2 minutes. Analyses of the initial contact angles for all potato cultivars revealed that only Lady Claire and Russet Burbank showed no statistically significant differences. In turn, the final contact angle values were not significantly different between Bryza and Sweet Caroline. In both cases, the droplets spread faster than the measurement could detect (water droplets spread over the leaf surface and complete wetting occurred).

The heterogeneity of the leaf surfaces, expressed as drop asymmetry (DA) indices and calculated in 2018 and 2019, along with the standard deviation, are summarized in Table 1. In 2018, the highest index values (the highest surface heterogeneity) were obtained for the Russet Burbank cultivar, both by analyzing the average index value calculated for 1 s of measurement (DAin), as well as by analyzing the average index value for all 120 recorded measurements (DA). The lowest DA indices values were obtained for the Bryza genotype, both by analyzing the results for 1 s of measurement and for all recorded images. In 2019, higher DAin values were observed for Bryza than for the Rudawa cultivar. However, DAin values were comparable for these cultivars.

### 2.2. Microscopic Leaf Analysis

**Figure 3:**
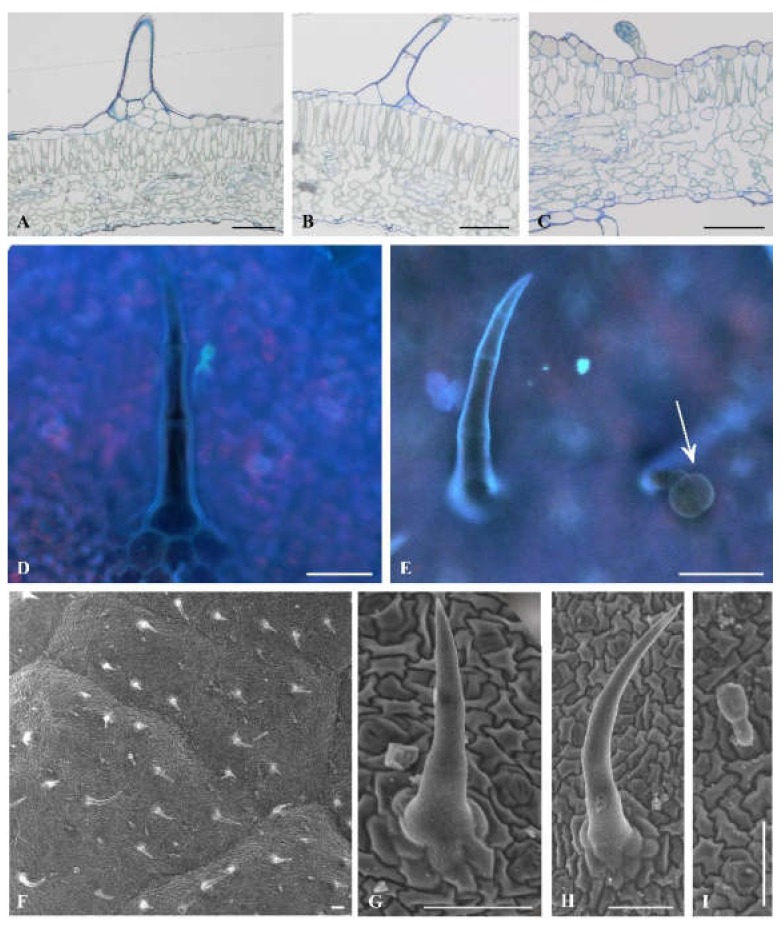
Trichome types on the Bryza adaxial leaf surface. Light microscopy views of leaf cross-sections (**A**–**C**). Epifluorescence (**D**, **E**) and SEM imaging: surface (**F**), trichomes (**G**−**I**). Non-glandular types: group II **A**, **D**, **G**, group V: **B**, **E**, **H**, glandular type VII present on **C**, arrow at **E** and **I**. All scale bars represent 100 µm.

**Figure 4:**
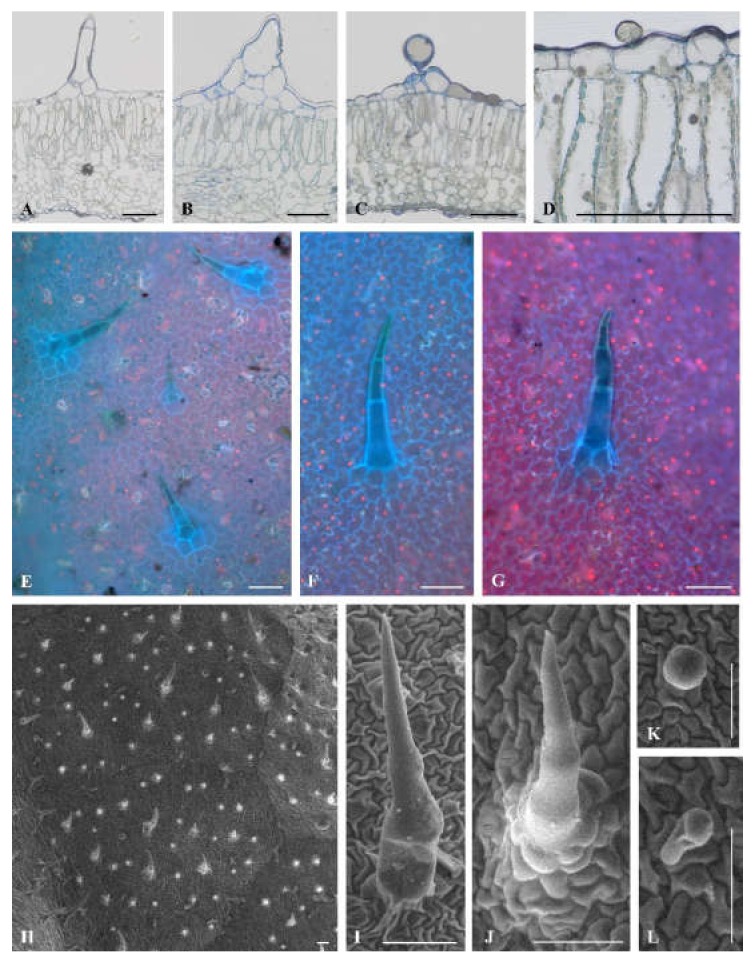
Trichome types on the Rudawa adaxial leaf surface. Light microscopy views of leaf cross-sections (**A**−**D**). Adaxial leaf surface at epifluorescence (**E**) and SEM (**H**), and trichomes (**F** and **G**) and (**H**−**L**), respectively. Non-glandular types: group II: **B**, **G**, **J**, group V: **A**, **F**, **I**, glandular types: group VII **C** and **L**, group VI **D** and **K**. All scale bars represent 100 µm.

Microscopic leaf analysis was limited only to cultivars Bryza and Rudawa, and this showed significant differences in the contact angle values. We did not observe any differences between Bryza and Rudawa cultivars (Figure 3A–C; Figure 4A–D) in the internal structure of the leaf blade. However, scanning electron microscopy (SEM) analysis indicated the diversity of trichome types on the leaf surface of the two exanimated potato cultivars. One type of glandular trichome (VII) and two types of non-glandular trichome (II and V) were visible on the Bryza leaf surface (Figure 3D–I), while in case of Rudawa (Figure 4E–L), two types of glandular trichome (VI and VII) and two types of non-glandular trichome (II and V) were observed. This means that the Rudawa leaf surface differed from Bryza through the presence of the glandular trichomes, type VI. The calculated value of trichome density for the Bryza cultivar was equal to 4.9 ± 1.9 pcs mm^−2^, in contrast to the Rudawa cultivar, for which the density was equal 14.8 ± 5.4 pcs mm^-2^. Trichome density differed between these two cultivars (*P* = 0.0387).

## 3. Discussion

Potato (*Solanum tuberosum*), from the Solanaceae family, is a crop which significantly contributes to meeting global food demand and trade, and has enormous economic benefits. Due to its considerable popularity, potato has a huge number of cultivars characterized by different growing seasons, tuber size and color, disease resistance, cultivation requirements, storage, and usability as well as morphology. Our research indicated that greater the resistance of a cultivar to biotic stress may be connected to the level of leaf wettability, which is a consequence of differences in the structure of the leaf epidermis. In this article, the leaf wettability of five potato cultivars was examined. The Rudawa cultivar was characterized by the highest contact angle values (62.4°), and the water droplet on the leaf behaved most stably during the measurement compared to the other cultivars. In turn, the lowest contact angle values were obtained for the Bryza cultivar (22.8°). These differences are probably related to the cultivars’ traits. Bryza belongs to the medium–late edible group, while Rudawa is a late starch cultivar. In addition, Rudawa is more resistant than Bryza to pests and pathogens [27].

Light microscope analysis showed no differences in the structure of the leaf blade between the two selected and examined cultivars (Figure 3 and Figure 4). However, on the epidermis surface, different types of hair were visible. Trichomes (also called hairs), are specialized epidermal structures that protect plants from abiotic and biotic stress conditions because they exist in direct contact with the surrounding environment. Trichomes have different structures and functions. Their location on plant organs, their size, and their density are variable between different species, and even within a species [28]. Based on trichome morphology, they can be divided into single-cell and multi-cell, branched or unbranched. Moreover, depending on the presence of glandular heads, trichome types of the tomato (*Solanum lycopersicum*) species, a native to the Solanaceae family, are divided into two distinct groups: glandular (types I, IV, VI, and VII) and non-glandular (types II, III, and V) [29]. Type I trichomes are characterized by their multicellular base, long multicellular stalk, and their small glandular head. Type IV trichomes have a unicellular base, a multicellular stalk, and a small secreting head. Type VI trichomes comprise a four-celled glandular head on a short multicellular stalk. Type VII trichomes consist of a short unicellular stalk and an eight-celled head [29]. Non-glandular type II and III trichomes are similar in shape, but differ in the nature of their base, which is multicellular or unicellular, respectively. Type V trichomes are shorter than type II and III trichomes, and have a unicellular base [29,30]. Four hair types of potato are very similar to tomato non-glandular (II and V) and glandular (VI and VII) trichomes. Like the cultivated potato species, the 17 wild potato species also possess four different trichome types, although the density of each trichome type displays considerable variation among the wild species. Cho et al. [29] divided the 17 examined species into four groups based on trichome density and type. Group I have more glandular trichomes compared to those in other species; group II have more non-glandular trichomes on both leaf surfaces; group III contains only one species, which has the highest density of abaxial leaf-surface non-glandular trichomes; and group IV species display non-glandular and glandular trichome density at an intermediate or lesser level compared to species in other groups.

Our measurements of contact angle values and SEM analyses of the potato leaf surfaces indicated that the level of wettability was closely related to the type of trichomes and their density. In the case of the Rudawa cultivar, we observed two types of glandular trichome, VI and VII, and two types of non-glandular trichome, II and V. In turn, on the Bryza leaf surface there were two non-glandular trichomes (as with the Rudawa cultivar), but only one type (VII) of glandular trichome. Non-glandular trichomes are a physical barrier against herbivory, while glandular trichomes are known to produce and secrete a wide variety of plant secondary compounds including terpenoids [31,32,33], phenolics [34], sucrose esters, methyl ketones [35], and organic acids [36]. Some of these metabolites are effective as a plant defense mechanism against herbivores and pathogens [37]. Type VI trichomes produce 2-tridecanone and other methylketones that are highly toxic to tomato pests [38]. Therefore, the greater resistance of the Rudawa cultivar to biotic stress conditions could be the result of the presence of two glandular trichome types (VI and VII) which produce and secrete metabolites containing various sticky and/or toxic chemicals that may poison or repel herbivores [33]. Furthermore, the trichome density of the Rudawa cultivar is higher than that of the Bryza cultivar. Traw and Bergelson [39] observed that jasmonic acid regulates trichome production and plant defense. Induction of resistance to herbivores and pathogens is generally regulated by a network of signal transduction pathways in which salicylic acid and jasmonic acid function as key signaling molecules [40,41,42]. Generally, jasmonic acids are a class of lipidic plant hormones involved in development, abiotic stress responses, and plant–microbe interactions in defense and symbiosis. Jasmonic acids are either methylated [43] or adenylated [44] during the process of induction, with different consequences for the expression of resistance. In the tomato, methyl jasmonate increases the density of glandular hairs on new leaves [45]. The induction of methyl jasmonate by herbivores and the subsequent hair density is ecologically significant because hair density can negatively affect herbivore populations [46,47,48]. According to Van Schie et al. [32], jasmonate might also induce trichome-based defenses directly. The production of acyl sugars on the leaf surface (probably in the trichomes) of *Datura wrightii* plants increased without affecting trichome density [32,49], and nornicotin production on the *Nicotiana repanda* plant surface increased 2-fold within 6 h of jasmonate treatment [50]. 

To clarify the role of potato cultivar trichomes for various species of herbivores and pathogens, it is necessary to analyze glandular metabolites and identify those metabolites that provide potato resistance to the various selected pests.

The obtained wetting contact angle values differed significantly between cultivars, and ranged from 22.8° for the Bryza cultivar to 62.4° for the Rudawa cultivar. In addition to the differences in the types of trichome on the leaf surfaces of the Bryza and Rudawa cultivars, the number of trichomes on the leaf surface was also different, as demonstrated by SEM analysis of the photos. For the Rudawa cultivar, the density of trichomes was more than three times that of the more wettable Bryza cultivar. Therefore, there may also have been different amounts of hydrophobic waxes on their leaf surfaces. Szafranek and Synak [51] showed that while the qualitative composition of waxes on leaves for different potato cultivars is similar, there may be quantitative differences between the cultivars. 

Potato (*Solanum tuberosum* L.) belongs to the family Solanaceae. In the same family, there are plants with similar values of leaf contact angle, e.g., ordinary tomato *Solanum lycopersicum*, with a contact angle of the upper surface of the leaf at 55.95°, or eggplant at 81.85° [26]. The higher contact angle values in the Rudawa cultivar mean that, in contrast to the Bryza cultivar, the water drops wet a smaller area of the leaf. This may be associated with the greater resistance of Rudawa to *Phytophthora infestans* (Table 2). Therefore, potato leaf wettability could be an important indicator for choosing cultivars that are the most resistant to pathogens, including the most important, namely *P. infestans*. Leca et al. [52] confirmed that for the apple leaf, wetness duration is a crucial variable in epidemiological risk assessment. They also observed that wettability could be a direct expression of the genetic resistance of apple genotypes to apple scab (*Venturia inaequalis* (Cooke) G. Winter), thus influencing the physicochemical properties of the surface.

## 4. Materials and Methods

Five potato cultivars were selected for leaf contact angle measurement analysis (see Table 2). The plants came from the Plant Collection of the Institute of Agriculture at Warsaw University of Life Sciences. Potatoes were grown on Luvisol with a loam texture. Potato crops were rainfed and non-pesticides were used to eliminate their potential impact on the leaf surface. The plant analyses were carried out in the summer of 2018, then in 2019 the same analyses were repeated for the two potato cultivars that showed the greatest differences in leaf wettability, i.e., Bryza and Rudawa. Potato leaves (terminal leaflets) were gently harvested at the tuber initiation growth stage, stored in a humid environment in a portable refrigerator, and quickly transported to the laboratory. All analyses were performed within two days of leaf collection.

### 4.1. Contact Angle

Contact angle measurements were made using the sessile drop method with an optical goniometer CAM 100 (KSV Instruments, Finland). The experiments were carried out in laboratory conditions (room temperature at about 20 °C and relative humidity ranging from 30% to 40%). For each potato cultivar, a few terminal leaflets were selected for analysis. Between two and four leaflets were analyzed. This leaflet number depended on the leaf size. Ten distilled water drops were placed on the leaves’ surfaces. During the replication of the contact angle measurements the water drop has never been placed on the same leaf part. Only the adaxial leaf part was included in the analyses. The camera built into the goniometer recorded the image of the water drops, and then the corresponding software aligned the droplet shape and measured the contact angle values on the left and right drop sides. Twenty measurements for each cultivar were taken. Drop behavior on the leaf surface was observed for two minutes and recorded for every consecutive second. The initial contact angle (t = 1 s), the final contact angle (t = 120 s), and the differences between initial and final contact angle were analyzed.

Statistical analyses using a two-way analysis of variance (ANOVA) in the Statgraphics Plus program for Windows 4.1 (STSC-Inc.-Statistical Graphics Corporation, 1996) were conducted for the contact angle results. The mean comparison was done using the LSD range test at *P* < 0.05.

The drop asymmetry (DA) index was calculated to estimate the heterogeneity of the leaf surface using the following equation [10]:

(1) $$\mathrm{DA}=2\cdot \left|\frac{{\mathrm{CA}}_{R}-{\mathrm{CA}}_{L}}{{\mathrm{CA}}_{R}+{\mathrm{CA}}_{L}}\right|$$

where CA_R_ is the contact angle measured on the right drop side and CA_L_ is the contact angle measured on the left drop side.

### 4.2. Morphological Leaf Analysis

Apical leaflet segments were dissected, fixed in 2% paraformaldehyde in a 0.1 M sodium cacodylate buffer for 2 h, washed four times in a 0.1 M sodium cacodylate buffer for 10 min at the room temperature, dehydrated, and embedded in an epoxy resin at 60 °C for 24 h according to the manufacturer’s formula. Light microscopy analyses were conducted on sections obtained from the same samples. Samples were serially sectioned using a RM2165 microtome (Leica Microsystems, Wetzlar, Germany) into 3 µm sections which were collected on glass slides, stained with an aqueous solution of crystal violet dye (1%, Sigma-Aldrich, St. Louis, MO, USA), and imaged on an AX70 Provis (Olympus, Tokyo, Japan) light microscope equipped with an Olympus DP50 digital camera (Olympus). 

The specimens for surface observations were collected from apical leaf segments, incubated in absolute ethanol for 1 min and placed on glass slides, and then examined using UV irradiation in epifluorescence on an AX70 Provis (Olympus) light microscope equipped with an Olympus DP50 digital camera and narrow-band filter set U-MNU (Olympus, Tokyo, Japan).

A scanning electron microscope (FEI Quanta 200 SEM, Hillsboro, OR, USA) was also used for analysis of the surface structures of the examined leaves. For the Bryza and Rudawa cultivars, trichome density, expressed in pcs per mm^2^, was determined on the basis of SEM images with a 70-fold magnification (three images for each species). The mean and standard deviation values were calculated. The trichome density results were tested for statistically significant differences, by using the t test. The differences with *P* values below 0.05 were treated as significant.

Digital images were adjusted for similar contrast and brightness, cropped, and resized using Adobe Photoshop software.

## 5. Conclusions

The results of contact angle measurements demonstrated variations of leaf wettability between potato cultivars. It was observed that the level of leaf wettability was connected to the cultivar resistance to biotic stress, which is a consequence of the differences in leaf epidermis structure. Among the five analyzed cultivars, Rudawa leaves exhibited the lowest wettability. The SEM image analysis indicated the presence of two types of glandular trichomes on Rudawa leaf surfaces, with a trichome density 3-fold greater than the most wettable Bryza cultivar. Probably as a consequence of this, Rudawa is a more resistant cultivar to pests and pathogens than Bryza. Since potatoes are one of the most important food crops in the worlds, leaf wettability of this crop could be an important indicator for choosing cultivars that are the most resistant to pathogens, including the most important one, namely *P. infestans.*

## Figures and Tables

**Table 1:** Drop asymmetry of potato leaf surface calculated for 1 s (DAin) and for all 120 s (DA) for the potato cultivars examined in the years 2018 and 2019.

Potato Cultivar	DAin	SDin	DAin	SDin	DA	SD	DA	SD
Year of research	2018		2019		2018		2019	
Bryza	0.085	0.112	0.123	0.110	0.086	0.091	0.138	0.110
Lady Claire	0.089	0.065	-	-	0.176	0.142	-	-
Rudawa	0.112	0.068	0.030	0.029	0.218	0.189	0.142	0.119
Russet Burbank	0.131	0.062	-	-	0.276	0.164	-	-
Sweet Caroline	0.098	0.158	-	-	0.145	0.147	-	-

**Table 2:** Potato cultivar characteristics.

Cultivar	Breeder/Country	Resistance to *P. Infestans* *	Foliage Cover	Time of Maturity	Use
Bryza	Pomorsko-Mazurska Hodowla Ziemniaka sp. z o.o., PL	medium	good	semi-late	table
Lady Claire	C. Meijer B.V., NL	very low to low	good to dense	early	table
Rudawa	Hodowla Ziemniaka Zamarte sp. z o.o. - Grupa IHAR, PL	medium to high	good	late	starch
Russet Burbank	GB Seed Industry, USA	low to medium	good to dense	very late	table
Sweet Caroline	Under registration medium	NA	NA	NA	NA

* Cultivar leaf resistance to Phytophthora infestans of the Polish origin is given according to the Research Centre for Cultivar Testing (RCCT); NA: data not available.
